# The effect of first trimester hemoglobin levels on pregnancy outcomes

**DOI:** 10.4274/tjod.87269

**Published:** 2018-09-03

**Authors:** Burcu Dinçgez Çakmak, Ülkü Ayşe Türker, Sonay Öztaş, Melis Arık, Emin Üstünyurt

**Affiliations:** 1 University of Health Sciences, Bursa Yüksek İhtisas Training and Research Hospital, Clinic of Obstetrics and Gynecology, Bursa, Turkey

**Keywords:** First trimester, hemoglobin, pregnancy outcome

## Abstract

**Objective:**

The relationship between hemoglobin levels and pregnancy outcomes is still a challenging issue. There is a supported opinion about the increased adverse pregnancy outcomes both with low and high hemoglobin levels. In this study, we aimed to evaluate this association for first trimester hemoglobin levels in a Turkish population.

**Materials and Methods:**

In this retrospective study, 1306 women who were followed up during their pregnancy and gave birth in our clinic were enrolled. The patients were divided into three groups: hemoglobin <11 g/dL (n=490), 11≤ hemoglobin <13 g/dL (n=673), and hemoglobin ≥13 g/dL (n=143). The hemoglobin <11 g/dL group was classified into two subgroups as hemoglobin ≤9 g/dL (n=64) and hemoglobin >9 g/dL (n=426). Demographic characteristics, first trimester hemoglobin levels, gestational age at delivery and mode, birth weight, Apgar scores, and pregnancy outcomes were recorded and compared between the groups.

**Results:**

Pregnancy-induced hypertension, preterm birth, neonatal intensive care unit admission, birth weight, gestational age at delivery, Apgar scores, and postpartum hemorrhage were significantly different between the three groups. In the pairwise comparison, gestational age at delivery, birth weight, and first minute Apgar scores were higher in the 11≤ hemoglobin <13 g/dL group, and pregnancy-induced hypertension was more common in the hemoglobin ≥13 g/dL group as compared with the others. Moreover, the preterm delivery rate was highest in the hemoglobin ≥13 g/dL (26.6%) group and lowest (7.3%) in the 11≤ hemoglobin <13 g/dL group. The neonatal intensive care unit admission rate was higher both the hemoglobin <11 g/dL and hemoglobin ≥13 g/dL groups. Postpartum hemorrhage was more common in the hemoglobin <11 g/dL group as compared with the other groups. Furthermore, pregnancy-induced hypertension was more common in the hemoglobin ≤9 g/dL subgroup (p=0.012).

**Conclusion:**

In conclusion, both low and high hemoglobin levels are related with adverse pregnancy outcomes. We suggest that hemoglobin levels must be screened during pregnancy to provide maternal and fetal well-being.

**PRECIS:** In this study, it is maintained that hemoglobin levels must be screened during pregnancy because both low and high hemoglobin levels are related with adverse pregnancy outcomes.

## Introduction

Pregnancy has various effects on hematologic parameters. It is well known that hemoglobin (Hb) levels decrease during the first trimester, reaching minimum values in the late second trimester and tend to increase during the third trimester of pregnancy^(1)^. Therefore cut-off levels for determining anemia differ from healthy reproductive women. The World Health Organization (WHO) defines anemia as Hb levels <11.0 g/dL in the first and third trimesters and <10.5 g/dL in the second trimester in pregnant women^(2)^. Recently, there was a supported opinion about the relationship between anemia and adverse pregnancy outcomes. Several studies claimed that maternal anemia was risk factor for adverse pregnancy outcomes such as low birth weight (LBW), postpartum hemorrhage (PPH), cesarean section (CS), and preterm birth (PB)^(3)^. Unfortunately, there are conflicting results about this issue. In another study, it was demonstrated that moderate or severe anemia was related with small-for-gestational-age infants, and patients with mild anemia were found to have uneventful pregnancy outcomes. Moreover, it was reported that preterm delivery and LBW were not increased in women with Hb 8-10.9 g/dL^(4)^. Another interesting finding of the recent studies is the risk of adverse pregnancy outcomes in pregnancies with high Hb levels. In pregnancy, both total erythrocyte number and plasma volume increase, but Hb levels decrease according to the higher increment in plasma volume. This condition provides placental perfusion with reduced blood viscosity^(5)^. A high Hb concentration during pregnancy could result in placental infarcts due to the increased viscosity. As a consequence, these pregnancies can be complicated with pregnancy-induced hypertension (PIH), fetal growth restriction, and perinatal death^(6)^. The association between Hb levels and adverse pregnancy outcomes differs by trimesters. However, it is more evident in early pregnancy for low Hb levels, and it is evident in all trimesters for high Hb concentrations^(7)^. There are limited data about the relationship between adverse pregnancy outcomes and Hb levels in the first trimester of pregnancy in the Turkish population. In the present study, we evaluated the effect of first trimester Hb levels on pregnancy outcomes in our population.

## Materials and Methods

This study was designed as a retrospective, observational, cross-sectional study. It was conducted in a university-affiliated training and research hospital between January 2016 and May 2017. Ethics committee approval is unnecessary for retrospective studies in our country. Our study complies with the Declaration of Helsinki. 

A total of 1306 women who were followed up during their pregnancy and gave birth in our clinic were enrolled in the study. First, patients were divided into three groups: Hb <11 g/dL (n=490), 11≤ Hb <13 g/dL (n=673), and Hb ≥13 g/dL (n=143). Then, the Hb <11 g/dL group was classified into two subgroups as Hb ≤9 g/dL (n=64) and Hb >9 g/dL (n=426). The exclusion criteria for patient selection were determined as follows; unregular antenatal visits, lack of delivery data, absence of first trimester Hb values, age <16 and >40 years, multiple pregnancies, congenital malformations, pregnancies with history of diabetes mellitus, hepatic and renal failure, thyroid diseases, any uterine malformations, previous complicated pregnancy, alcohol and cigarette use, any prior placental abnormalities and PPH. Demographic characteristics of the study population such as age, gravida, parity, and gestational age at delivery and birth weight were recorded. Also, first trimester Hb levels were obtained from medical records. In our hospital, Hb levels are determined using a Coulter LH780 Analyzer (Beckman Coulter Ireland Inc, Mervue, Galway, Ireland). Pregnancy outcomes were compared between the groups. The main perinatal outcomes were accepted as stillbirth, gestational diabetes mellitus (GDM), PIH, PB, neonatal intensive care unit (NICU) admission, PPH, CS, and low Apgar scores, and these outcomes were obtained from hospital records. Stillbirth was judged as the death of a fetus during delivery^(8)^. GDM was established as if one or more of the followings were increased in the 75 g oral glucose tolerance test: fasting glucose ≥92 mg/dL, ≥180 mg/dL at 1 hour, and ≥153 mg/dL at 2 hours^(9)^. PIH was named as systolic blood pressure ≥140 mm Hg and/or diastolic pressure ≥90 mm Hg after the 20^th^ gestational week, and PB was defined as births that occured between the 24-37^th^ gestational weeks^(10,11)^. Neonates with shorter than 32 weeks of gestation, transient problems, cardiorespiratory monitoring requirement or presence of Respiratory Distress syndrome, severe jaundice, neonatal sepsis, and conditions requiring exchange transfusion were admitted to the NICU. PPH was defined as having a blood loss of ≥500 mL after vaginal delivery or ≥1000 mL after CS within 24 hours of delivery^(12)^.

### Statistical Analysis

Statistical analyses were performed using the Statistical Package for the Social Sciences statistical software version 23.0 (SPSS, Chicago, IL). All data are reported as mean ± standard deviation, median [minimum (min), maximum (max)] values or in percentages. The Shapiro-Wilk test and probability plots were used to evaluate whether the variables followed normal distribution. The chi-square test and Fisher’s exact test were performed to evaluate the relationship between categorical variables. According to the normality test results, the Mann-Whitney U test was used for continuous non-normally distributed variables, and the independent t-test was used for continuous normally distributed variables to compare the variables between two groups. For comparing more groups, the non-parametric Kruskal-Wallis test was performed, and the Bonferroni-Dunn procedure was used to compare statistically significant parameters between two groups. Moreover, for normally distrubuted variables, one-way ANOVA analysis was performed to compare the variables between more than two groups. A p value of ≤0.05 was determined as statistically significant.

## Results

The mean age of all study participants was 27.24±6.21 years. The median gravida was 2 (min=1, max=10) and parity was 1 (min=0, max=8). The mean gestational age at delivery was 38.16±2.37 weeks and the mean birth weight was 3134.78±600.96 grams. The demographic data and pregnancy outcomes of three main groups were compared and are presented in Table 1. PIH, PB, NICU admission, birth weight, gestational age at delivery, Apgar scores, and PPH were significantly different between the three groups. In the pairwise comparison; gestational age at delivery, birth weight, and first-minute Apgar scores were significantly higher in 11≤ Hb <13 g/dL group, and PIH was more common in the Hb ≥13 g/dL group as compared with the other groups. Moreover, the PB rate was highest in the Hb ≥13 g/dL (26.6%) group and was lowest (7.3%) in the 11≤ Hb <13 g/dL group. The NICU admission rate was significantly higher in both the Hb <11 g/dL and Hb ≥13 g/dL group as compared with the 11≤ Hb <13 g/dL group. Furthermore, PPH was significantly more common in the Hb <11 g/dL group as compared with the other groups. The comparison of demographic characteristics and pregnancy outcomes between the Hb ≤9 g/dL and 9< Hb <11 g/dL group is shown in Table 2. There was no difference between the two groups according to gravida, parity, age, gestational age at delivery, birth weight, Apgar scores, stillbirth, GDM, PB, CS, NICU admission, and PPH rates. Contrary to these, PIH was significantly higher in the Hb ≤9 g/dL group (p=0.012).

## Discussion

The main findings of the study were as follows: PIH was more common in the high Hb group, and the incidences of PB and NICU admission were higher both in the high and low Hb groups. Moreover, PPH was common in the low Hb group. Gestational age at delivery, birth weight, and first-minute Apgar scores were significantly higher in the 11≤ Hb <13 g/dL group, and there was no difference according to pregnancy outcomes between the very low and low Hb groups, except PIH. During pregnancy, many hormonal changes occur to provide adequate blood flow from the maternal to the fetal unit. One of these changes is increased plasma renin and decreased atrial natriuretic peptide levels. Also, erytropoetin secretion tends to increase and results in a rise in red blood cell mass. On the other hand, plasma volume expands nearly 50% and consequently, Hb levels decrease. Disturbance of these mechanism leads to hemoconcentration and high Hb levels^(13,14)^. PIH is still an important cause of maternal and fetal mortality and morbidity. Although the underlying mechanism has not been fully elucidated, recent studies have shown that increased Hb levels leading to vasoconstriction is one of the mechanisms of PIH^(15)^. Also, the loss of protein and increment in vascular permeability causes a decrement in intravascular volume and high Hb concentrations in preeclampsia^(16)^. In a study by Pritchard et al.,^(17)^ the average hematocrit was higher in preeclampsia as compared with healthy pregnant women. In other studies, a significant relationship between high first-trimester Hb levels and preeclampsia was demonstrated^(18,19)^. Similarly, in this present study, PIH was more common with high Hb levels. We and others suggest that the changes of hematologic changes in PIH start early in the first trimester and monitoring Hb levels could be used to follow up pregnancies at high risk for uteroplacental insufficiency.^(20)^ Recent studies evaluating the PB risk in pregnant women in relation to Hb levels had conflicting results. Scanlon et al.,^(13) ^who divided the patient group into 7 levels as very low, low, low-normal, normal (reference group), high-normal, high, and very high Hb groups showed that patients with a first-trimester Hb concentration below the reference range had an elevated risk of PTB. Furthermore, in a study of a Chinese population, elevated PB risk was found in the low first-trimester Hb group^(21,22)^. On the other hand, no relationship was found between PB and first-trimester Hb levels in the study of Hamalainen et al.^(23)^. For high Hb levels, Zhang et al.^(22)^ found reduced risk for PB, and other studies claimed that no association was present between PB and high Hb levels.^(13,24)^ Zhou et al.^(25) ^reported slightly increased risk for PB with high Hb levels. In this present study, PB rates were higher with both low and high Hb levels. Moreover, NICU admission rates were higher in both the high and low Hb groups, which could be related to prematurity and accompanying conditions. PH is one of the leading causes of maternal mortality. Oxygen and Hb transportation is the cornerstone of uterine contractions and it is claimed in the literature that anemic patients were more likely to experience uterine atony due to the absence of these mechanisms^(26,27)^. In the study of Sehgal et al.,^(4)^ pregnant women with mild-to-moderate anemia were found to have more PPH. Similar to their study, we demonstrated that patients with Hb <11 g/dL had more PPH as compared with those in the 11≤ Hb <13 g/dL and Hb ≥13 g/dL groups. There is no consensus about iron replacement, to whom and how many milligrams should be given in pregnancy. The WHO recommends 30-60 mg daily iron during pregnancy. These data were based on the reduced risk of LBW with daily iron supplementation, increased risk for adverse effects, and adverse pregnancy outcomes for high Hb levels^(28,29)^. Supporting these recommendations, we found higher birth weights and Apgar scores, and lower PB in the 11≤ Hb <13 g/dL group and we suggest that appropriate Hb levels with iron supplementation must be constituted to provide maternal and fetal well-being. However, we did not investigate the effects of iron supplementation on our pregnancy outcomes, which is one of the major limitations of our study. Another interesting finding of our study was that there was no difference with regard to pregnancy outcomes between the very low and low Hb groups, except PIH. A few studies that investigated the effects of severe or moderate anemia demonstrated that patients with moderate and severe anemia were more prone to uterine atony and PPH^(26,27)^. Pregnant women with mild anemia are generally expected to have uneventful pregnancies if well managed with iron supplementation^(4)^. Another study showed that Hb 8-10.9 g/dL was not associated with an increased PB and LBW risk^(22,30)^. We suggest that our non-significant results between the very low and low Hb groups might be related to the small patient population with severe anemia.

### Study Limitation

This study has several limitations. First, we did not investigate the effects of iron supplementation on our pregnancy outcomes. Second, we had small patient population with very low Hb levels to compare the pregnancy outcomes between low and very low Hb levels. Lastly, we only evaluated the first trimester Hb levels and it might be more appropriate to clarify the relationship between all trimester Hb levels and pregnancy outcomes.

## Conclusion

In conclusion, both low and high Hb levels are related with adverse pregnancy outcomes. To provide maternal and fetal well-being, we must routinely screen the first trimester Hb levels and think about supplementing iron if it is appropriate.

## Figures and Tables

**Table 1:**
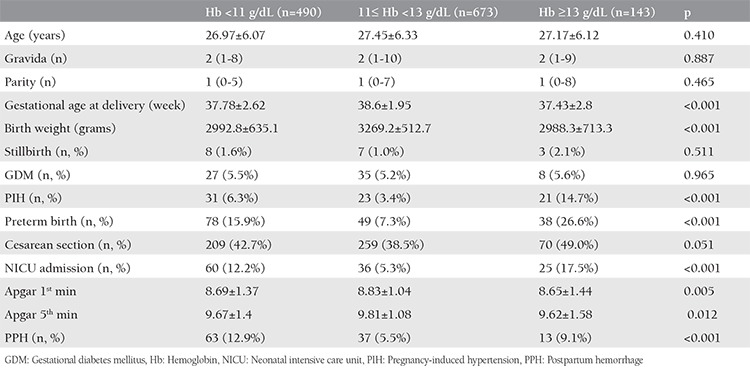
Demographic characteristics and pregnancy outcomes of all study groups

**Table 2:**
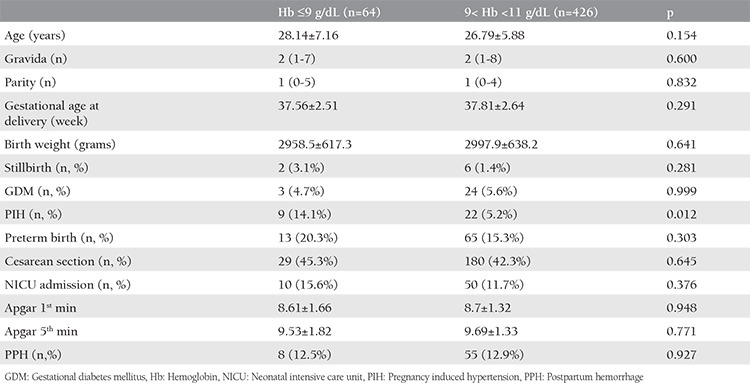
Demographic characteristics and pregnancy outcomes of Hb ≤9 g/dL and 9< Hb <11 g/dL groups
